# Pancreatic solid pseudopapillary neoplasm mimicking a pseudocyst in a young female with acute pancreatitis

**DOI:** 10.1002/jpr3.70166

**Published:** 2026-03-19

**Authors:** Shani Litwin, Sara K. Yang, Howard C. Jen, Alireza Sedarat, Alvin P. Chan

**Affiliations:** ^1^ Department of Pediatrics Harbor‐UCLA Medical Center Torrance California USA; ^2^ Department of Radiology UCLA Ronald Reagan Medical Center Los Angeles California USA; ^3^ Division of Pediatric Surgery, Department of Surgery UCLA Ronald Reagan Medical Center Los Angeles California USA; ^4^ Division of Digestive Diseases, Department of Medicine Ronald Reagan UCLA Medical Center Los Angeles California USA; ^5^ Division of Pediatric Gastroenterology, Department of Pediatrics UCLA Mattel Children's Hospital Los Angeles California USA

**Keywords:** abdominal mass, abdominal pain, Frantz tumor, surgical resection

## Abstract

Solid pseudopapillary epithelial neoplasm (SPEN) of the pancreas is a rare, low‐grade pancreatic neoplasm that is uncommon in the pediatric population. We present the case of previously healthy 11‐year‐old girl with a pancreatic mass initially misdiagnosed as a pseudocyst, later confirmed to be a SPEN. This report highlights key clinical and imaging features distinguishing SPEN from pancreatic pseudocysts and underscores the importance of maintaining a broad differential diagnosis when the clinical course is atypical.

## 1 INTRODUCTION

Pancreatic tumors in children are exceedingly rare, with an incidence of 0.02 per 100,000 children per year.^1^, ^2^ Among them, solid pseudopapillary epithelial neoplasm (SPEN) of the pancreas is the most common type in post‐pubertal children, occurring predominantly in females.^1^, ^3^ The ratio of female to male of 5:1 suggests a hormonal influence, although the exact etiology remains unclear.^4^ Clinically, SPENs can be difficult to identify due to non‐specific symptoms and a range of imaging features, which may lead to diagnostic confusion and delayed treatment.^1^ This report highlights key clinical and imaging characteristics that help distinguish SPENs from other pancreatic lesions.

## 2 CASE REPORT

**Figure 1:**
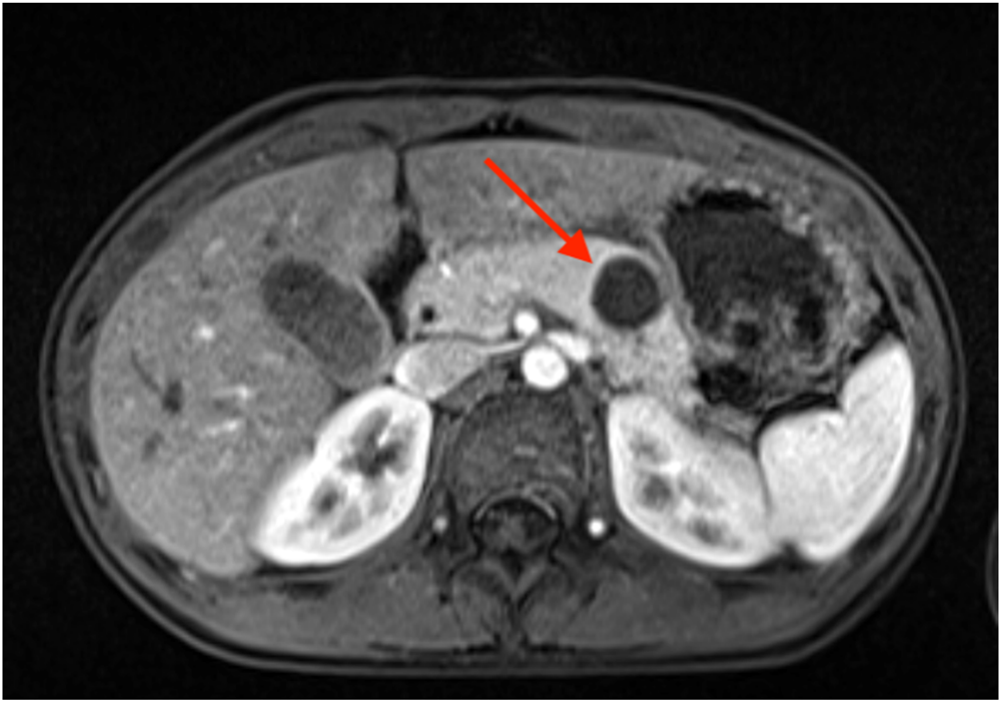
Axial fat‐saturated T1 post‐contrast magnetic resonance imaging (MRI) demonstrates a 1.7 × 1.7 cm well‐circumscribed non‐enhancing complex cystic structure within the pancreatic tail.

A previously healthy 11‐year‐old girl presented to the emergency department with worsening abdominal pain and vomiting. For the preceding month, she had experienced intermittent periumbilical pain, which acutely progressed to sudden‐onset, sharp abdominal pain, nausea, and vomiting on the day of presentation. Lab evaluation was significant for elevated amylase and lipase, consistent with acute pancreatitis. Ultrasound revealed a hypoechoic, avascular pancreatic tail lesion that was confirmed on magnetic resonance imaging (MRI), which demonstrated a well‐encapsulated, complex cystic lesion in the pancreatic tail (Figure 1). The lesion was presumed to be a pseudocyst. She was admitted to the hospital for fluid resuscitation with resolution of symptoms within 3 days. Following discharge, serial ultrasound imaging over the next 2 years demonstrated an interval decrease in the size of the lesion from 1.7 to 0.4 cm in diameter. She was then lost to follow up.

**Figure 2:**
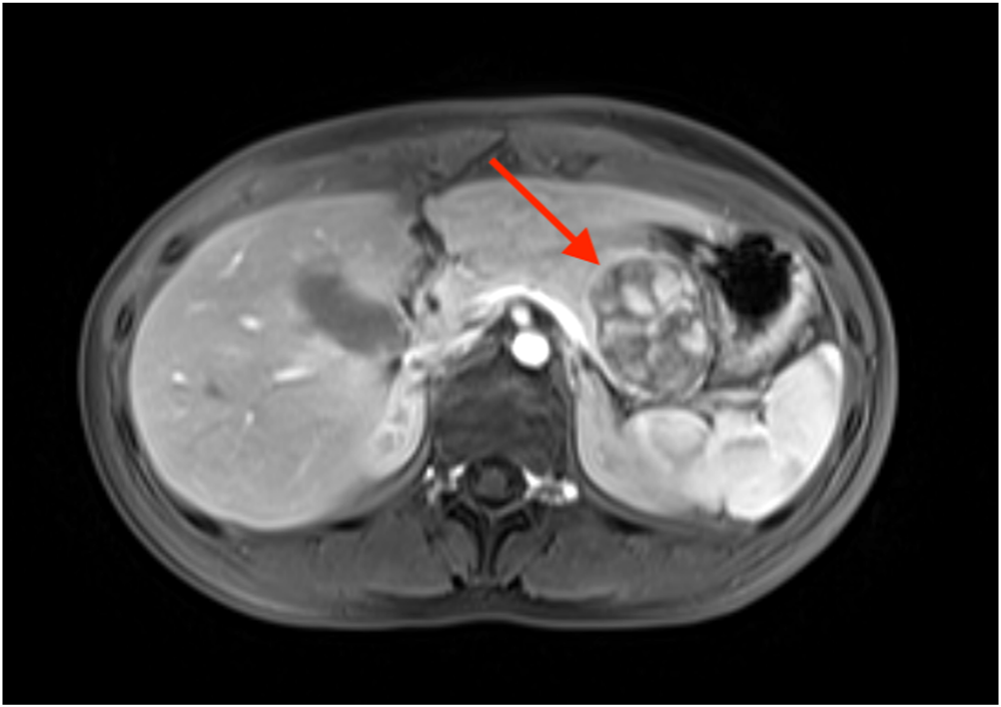
Axial fat‐saturated T1 post‐contrast magnetic resonance imaging (MRI) demonstrates a 4.5 × 3.8 cm well‐circumscribed mildly enhancing heterogeneous mass with internal papillary projections within the pancreatic tail.

After 4 years without symptoms, the patient, now 15 years old, re‐presented to her pediatrician with abdominal pain. Given the recurrence of symptoms, a repeat ultrasound was performed and showed enlargement of the same lesion. MRI demonstrated a well‐circumscribed heterogeneous mass with new enhancing solid components, raising concern for a neoplasm rather than a pseudocyst (Figure 2). Lipase, carbohydrate antigen 19‐9 (CA 19‐9), and carcinoembryonic antigen (CEA) were normal. A spleen‐preserving distal pancreatectomy was performed for diagnosis and treatment. Pathology revealed a multiloculated 4.2 cm cystic lesion containing abundant hemorrhagic fluid and areas of focal necrotic and fibrotic material with obliteration of the main pancreatic duct. The lesion was confined to the pancreas, and its edges had focal solid to papillary regions with negative margins. Immunohistochemistry staining was positive for several tumor markers, including *SOX11*, *CD10*, and *INSM1*. β‐Catenin showed a patchy nuclear staining pattern, reflecting aberrant Wnt/β‐catenin pathway activation. Taken together, the histological staining profile confirmed the diagnosis of SPEN.

## 3 DISCUSSION

Although pancreatic tumors are rare in children, SPEN is the most common pancreatic tumor in children and adolescents.^5^ Diagnosis is often delayed due to vague symptoms and the lack of reliable biomarkers. By the time of diagnosis, tumors are typically large, with median diameters between 5 and 8 cm.^6^ In children, SPENs are more frequently located in the head of the pancreas, whereas body and tail involvement are more common in adults.^3^

SPENs are low‐grade malignant tumors, making surgery both diagnostic and curative.^2^, ^4^ Complete surgical resection is the definitive treatment, with 5‐year survival rates exceeding 95%.^3^ Preoperative biopsy is usually deferred due to low sensitivity of fine‐needle aspiration. Consistent with adult recommendations,^7^ post‐surgical surveillance with annual imaging for at least 5 years is recommended to monitor for recurrence.^4^ In pediatric patients, recurrence rates are generally below 7% and are most strongly linked to incomplete surgical resection or intraoperative tumor rupture.^3^

Proper imaging is central to the diagnosis and evaluation of SPEN. Among the available modalities, MRI is most effective for detailed characterization. While pancreatic pseudocysts are usually unilocular, fluid‐filled lesions without solid elements, SPENs often appear as well‐circumscribed, heterogeneous masses composed of both solid and cystic components, often surrounded by a fibrous capsule.^1^, ^6^, ^7^, ^8^ Other distinguishing features include areas of calcifications or internal hemorrhage with pseudopapillary patterns or pseudorosettes.^6^ Ultrasound is useful for initial detection but is less specific than MRI for detailed characterization. In this case, although the surveillance ultrasounds suggested gradual regression, it likely failed to detect evolving solid components. Given their shared cystic features, SPENs can occasionally be mistaken for pancreatic pseudocysts.^8^ A similar misdiagnosis was reported in a 22‐year‐old woman who underwent exploratory laparotomy and biopsy of the cyst wall before the diagnosis was confirmed histologically.^9^ To our knowledge, no such cases have been reported in pediatric patients, likely reflecting the rarity of this tumor in the population.

Several clinical clues in this case warranted reconsideration of the differential diagnosis. The patient's acute pancreatitis episode was incorrectly attributed to the presumed pseudocyst; however, pseudocysts typically develop weeks after pancreatitis, not before. Thus, the lesion was more likely the cause rather than a complication of the acute episode. A diagnosis of pseudocyst without a preceding history of pancreatitis should be made cautiously. Clinicians should therefore maintain a high index of suspicion for pancreatic neoplasms, particularly when the clinical course is atypical, as early recognition leads to timely surgical resection, preventing unnecessary symptoms and complications.^5^

## 4 CONCLUSION

This case highlights the importance of careful assessment of atypical pancreatic lesions in pediatric patients. Recognizing key imaging features such as solid components and interval growth, alongside a critical assessment of the clinical course, can ensure early diagnosis and intervention. Heightened awareness of SPEN may improve diagnostic accuracy and ultimately patient outcomes.

## CONFLICT OF INTEREST STATEMENT

The authors declare no conflict of interest.

## ETHICS STATEMENT

The parents of the child in question are aware of this report and have given their consent to write this report and to include the images.

## References

[1] Patterson KN , Trout AT , Shenoy A , Abu‐El‐Haija M , Nathan JD . Solid pancreatic masses in children: a review of current evidence and clinical challenges. Front Pediatr. 2022;10:966943. 36507125 10.3389/fped.2022.966943 PMC9732489

[2] Mylonas KS , Doulamis IP , Tsilimigras DI , et al. Solid pseudopapillary and malignant pancreatic tumors in childhood: a systematic review and evidence quality assessment. Pediatr Blood Cancer. 2018;65(10):e27114. 10.1002/pbc.27114 29697193

[3] Bender AM , Thompson ED , Hackam DJ , Cameron JL , Rhee DS . Solid pseudopapillary neoplasm of the pancreas in a young pediatric patient. Pancreas. 2018;47(10):1364‐1368. 10.1097/mpa.0000000000001183 30325866

[4] Irtan S , Galmiche‐Rolland L , Elie C , et al. Recurrence of solid pseudopapillary neoplasms of the pancreas: results of a nationwide study of risk factors and treatment modalities. Pediatr Blood Cancer. 2016;63(9):1515‐1521. 10.1002/pbc.25986 27186826

[5] Gonda TA , Cahen DL , Farrell JJ . Pancreatic cysts. N Engl J Med. 2024;391(9):832‐843. 39231345 10.1056/NEJMra2309041

[6] Kim MS , Park H , Lee S , et al. Clinical characteristics, treatment outcomes, and occurrence of diabetes mellitus after pancreatic resection of solid pseudopapillary tumor in children and adolescents: a single institution experience with 51 cases. Pancreatology. 2021;21(3):509‐514. 33509684 10.1016/j.pan.2021.01.009

[7] Elta GH , Enestvedt BK , Sauer BG , Lennon AM . ACG clinical guideline: diagnosis and management of pancreatic cysts. Am J Gastroenterol. 2018;113(4):464‐479. 10.1038/ajg.2018.14 29485131

[8] Macari M , Finn ME , Bennett GL , et al. Differentiating pancreatic cystic neoplasms from pancreatic pseudocysts at MR imaging: value of perceived internal debris. Radiology. 2009;251(1):77‐84. 19332847 10.1148/radiol.2511081286

[9] Patel VG , Fortson JK , Weaver WL , Hammami A . Solid‐pseudopapillary tumor of the pancreas masquerading as a pancreatic pseudocyst. Am Surg. 2002;68(7):631‐632. 12132748

